# Foreign body granuloma in the anterior abdominal wall mimicking an acute appendicular lump and induced by a translocated copper-T intrauterine contraceptive device: a case report

**DOI:** 10.1186/1752-1947-3-7007

**Published:** 2009-04-03

**Authors:** Maulana Mohammed Ansari, Syed Hasan Harris, Shahla Haleem, Rehan Fareed, Mohammed Feroz Khan

**Affiliations:** 1 Department of Surgery, Jawaharlal Nehru, Medical College Hospital, A.M.U., Aligarh, U.P., India; 2 Department of Anaesthesiology, Jawaharlal Nehru, Medical College Hospital, A.M.U., Aligarh, U.P., India

## Abstract

**Introduction:**

Intrauterine contraceptive devices may at times perforate and migrate to adjacent organs. Such uterine perforation usually passes unnoticed with development of potentially serious complications.

**Case presentation:**

A 25-year-old woman of North Indian origin presented with an acute tender lump in the right iliac fossa. The lump was initially thought to be an appendicular lump and treated conservatively. Resolution of the lump was incomplete. On exploratory laparotomy, a hard suspicious mass was found in the anterior abdominal wall of the right iliac fossa. Wide excision and bisection of the mass revealed a copper-T embedded inside. Examination of the uterus did not show any evidence of perforation. The next day, the patient gave a history of past copper-T Intrauterine contraceptive device insertion.

**Conclusions:**

Copper-T insertion is one of the simplest contraceptive methods but its neglect with inadequate follow-up may lead to uterine perforation and extra-uterine migration. Regular self-examination for the "threads" supplemented with abdominal X-ray and/or ultrasound in the follow-up may detect copper-T migration early. To the best of our knowledge, this is the first report of intrauterine contraceptive device migration to the anterior abdominal wall of the right iliac fossa.

## Introduction

Increased patient acceptance of intrauterine contraceptive devices (IUCD), especially copper-T, without proper follow-up is associated with many early and late complications, including perforation and migration into adjacent structures in 1/350 to 1/2500 cases [1]. Migration of IUCDs into the urinary bladder, rectum, colon, peritoneum, omentum, appendix, wall of the iliac vein and ovary has been reported [2]. Herein we report the first case of IUCD migration to the anterior abdominal wall in the right iliac fossa (RIF) with foreign body granuloma formation, mimicking an acute appendicular lump.

## Case presentation

A 25-year-old woman was referred to us with a 5 day history of moderate localized pain in her right lower abdomen that was not radiating to any other site and was not associated with nausea or vomiting. The patient had mild pyrexia (temperature 99.4°F). On examination of her abdomen, a well-defined mildly tender localized fixed lump 7×5cm in size was found in the right iliac fossa. The hemogram showed a total leukocyte count of 11,000/mm^3^, with 60% polymorphonucleocytes. Ultrasonography (USG) of her abdomen revealed an oval-shaped abdominal mass in the right iliac fossa, suggestive of an appendicular lump.

The patient was put on the Ochsner-Sherren regimen. However, recovery was found to be slow and incomplete, and a smaller non-tender lump 5×5cm in size was still present at the end of 4 weeks. Repeat USG was suggestive of an unresolved appendicular lump.

On exploratory laparotomy through a lower midline incision, a hard mass lesion was found on the inner side of the anterior abdominal wall of the right iliac fossa, to which omentum was firmly adherent. The appendix was found to be normal and a wide-based Meckel's diverticulum was also present at 2 feet proximal to the ileo-caecal junction. Wide excision of the suspicious lesion was carried out with a clearance margin of 2cm all round and the resultant fascio-muscular defect in the anterior abdominal wall was repaired with polypropylene mesh. The Meckel's diverticulum and the normal appendix were also excised.

**Figure 1:**
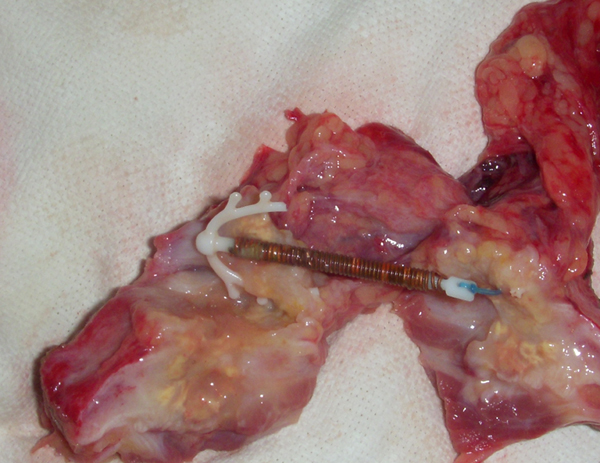
**Copper-T in the excised transected mass**.

The excised mass was bisected and, to our surprise, a copper-T IUCD was found embedded inside (Figure 1). The uterus was examined but there was no evidence of any perforation. The abdomen was closed and a tube drain was left in situ.

On cross-checking with the patient on the following day, she gave a history of copper-T insertion about 6 months previously.

The drain was removed after 48 hours, and the postoperative period was uneventful. The patient was discharged from the hospital on the 7th day after removal of stitches. She was asymptomatic at 1-month follow-up.

## Discussion

Since their introduction in 1965, intrauterine contraceptive devices (IUCD) are commonly used as an effective, safe and economic method of long-term contraception. Translocation of an intrauterine contraceptive device to an extra-uterine site is an uncommon but potentially serious complication but this may remain asymptomatic or present with varying abdominal symptoms and signs, depending on the severity of involvement [2]. Migration to the urinary bladder is commonly reported [3]; however, a migrated copper-T has also been recovered from the rectum [4] and from the sigmoid colon [5]-[7]. Up to 2005, 15 cases of acute appendicitis induced by migrated IUCD have been reported [8]. To the best of our knowledge, this is the first report of IUCD migration to the anterior abdominal wall of the right iliac fossa.

In cases reported in the literature, the timing of extra-uterine presentation and the distant sites of translocation often raise the issue of whether iatrogenic uterine perforation or migration of the device was responsible. Primary iatrogenic uterine perforation usually occurs at the time of IUCD insertion but an IUCD may become embedded in the uterus and later be forced through the wall by spontaneous uterine contractions [9]. However, other possible translocatory mechanisms such as urinary bladder contractions, gut peristalsis and movement of peritoneal fluid may also play a significant role [10]. Factors contributing to the possibility of uterine perforation are inept insertion or positioning, fragility of the uterine wall due to recent birth, abortion or pregnancy in general. Chang and colleagues [8] also emphasized that the incidence is influenced by factors such as the timing of insertion, parity, type of IUD inserted, experience of the operator and position of the uterus. Increased risk of IUCD translocation has also been observed in lactating mothers [11].

A translocated IUCD induces a dense fibroblastic reaction [11] which is the usual cause of it occasionally not being detected on ultrasonography, as was the case in our patient, or routine laparoscopy [2,12]. Hence, plain X-ray of abdomen and pelvis, the classical routine investigation, but nowadays often forgotten in the heat of freely available ultrasounds and contrast enhanced computed tomography (CT) scans, appears to be more the reliable method, as has been emphasized by Katara and colleagues [2].

## Conclusions

Uterine perforation and migration of IUCD usually passes unnoticed. Therefore, regular self-examination for "missing threads" supplemented with clinico-radiological controls in the follow-up after IUCD insertion can detect these migrations early. Easily available plain X-ray of abdomen and pelvis may be the simplest tool for early detection of a migrated IUCD and thereby avoid diagnostic difficulties and potentially serious complications.

## Consent

Written informed consent was obtained from the patient for publication of this case report and any accompanying images. A copy of the written consent is available for review by the Editor-in-Chief of this journal.

## Competing interests

The authors declare that they have no competing interests.

## Authors' contributions

MMA was in charge of the overall care of the patient and researched the literature and prepared the manuscript, with RF and MFK involved in follow-up care and manuscript preparation. SH was solely responsible for anesthesia and postoperative recovery. Critical review and submission was carried out by SHH. All five authors read and approved the final manuscript.

## References

[B1] Ohana E Sheiner E Leron E Mazor M Appendix perforation by an intrauterine contraceptive device Eur J Obstet Gynecol Reprod Biol 2000 88 129 131 10.1016/S0301-2115(99)00142-6 10690669

[B2] Katara AN Chandiramani VA Pandya SM Nair NS Migration of intrauterine contraceptive device into the appendix Indian J Surg 2004 66 179 180

[B3] Sasidharan K Chally R Intravesical migration of Lippes loop with stone formation Br J Urol 1998 61 363 364 10.1111/j.1464-410X.1988.tb13980.x 3289679

[B4] Laxami M Hemlata J Rani LP An unusual case of copper-T in the rectum J Obstet Gynecol India 2005 55 79 80

[B5] Browning JJ Bigrigg MA Recovery of the intrauterine contraceptive device from the sigmoid colon. Three case reports Br J Obstet Gynaecol 1988 95 530 532 3042019 10.1111/j.1471-0528.1988.tb12813.x

[B6] Nceboz AES Zakir HTA Uyar Y Ayar YH Migration of an intrauterine contraceptive device to the sigmoid colon: a case report Eur J Contracept Reprod Health Care 2003 8 229 232 10.1080/713604475 15006271

[B7] Mansoor T Aslam M Rizwi SAA Haseen MA Copper-T causing perforation of sigmoid colon Internet J Surg 2007 13

[B8] Chang HM Chen TW Hsieh CB Chen CJ Yu JC Liu YC Shen KL Chan DC Intrauterine contraceptive device appendicitis: A case report World J Gastroenterol 2005 11 5414 5415 16149161 10.3748/wjg.v11.i34.5414 PMC4622824

[B9] Carson SA Gatlin A Mazur M Appendiceal perforation by Copper-7 intrauterine contraceptive device Am J Obstet Gynecol 1981 141 586 587 7294085 10.1016/s0002-9378(15)33284-1

[B10] Eke N Okpani AOU Extra-uterine translocated contraceptive device: A presentation of five cases and revisit of the enigmatic issues of iatrogenic perforation and migration Afr J Reprod Health 2003 7 117 123 10.2307/3583296 15055154

[B11] Mittal S Gupta I Lata P Mahajan U Gupta AN Management of translocated and incarcerated intrauterine contraceptive devices Aust NZ J Obstet Gynaecol 1986 26 232 234 10.1111/j.1479-828X.1986.tb01574.x 3468943

[B12] Kriplani A Garg P Sharma M Agarwal N Laparoscopic removal of extra-uterine IUCD using fluoroscopy guidance: a case report J Gynecol Surg 2005 21 29 30 10.1089/gyn.2005.21.29

